# Supplementary material for: Diversity and Distribution of Hydrocarbon-Degrading Genes in the Cold Seeps from the Mediterranean and Caspian Seas
Source: Microorganisms. 2025 Jan 21;13(2):222. doi: 10.3390/microorganisms13020222 (PMC11857318; doi:10.3390/microorganisms13020222)
Supplement: Supplementary file 1 [file microorganisms-13-00222-s001.zip › Yogita_Et_al_MDPI_Supplemental_Information.pdf]

Supplemental Information:

16S Sequencing Results :

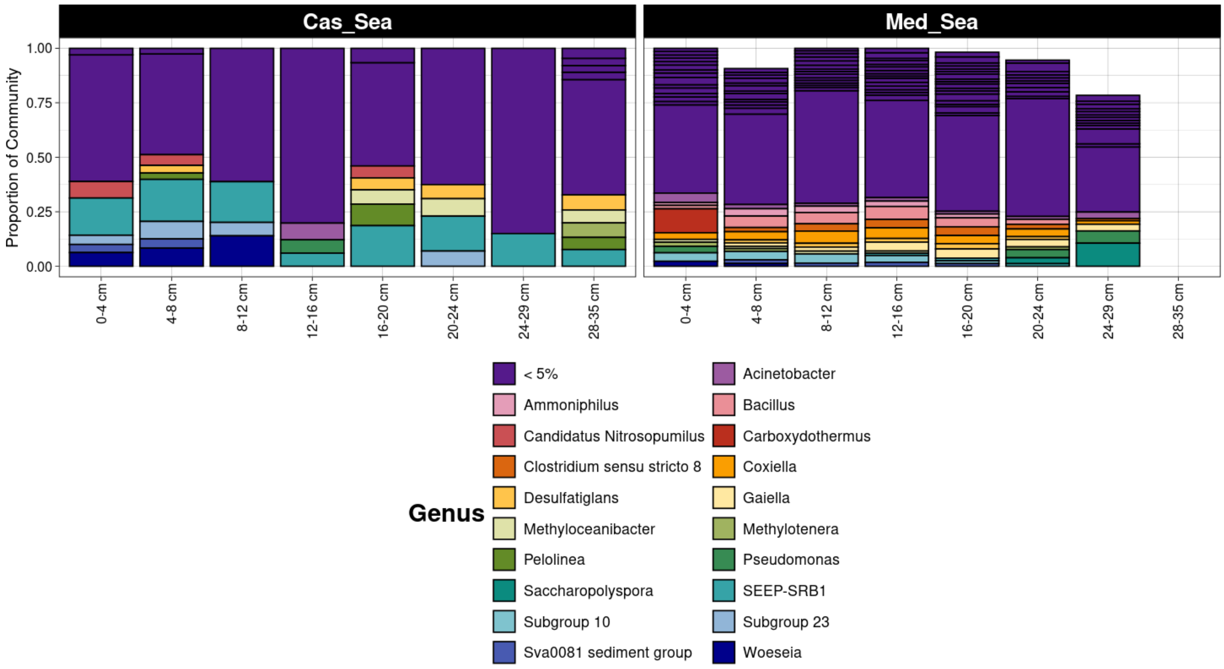

Figure S1: Microbial Community Composition at the Genus Level in Caspian and Mediterranean Sea Sediments Based on 16S rRNA Sequencing.

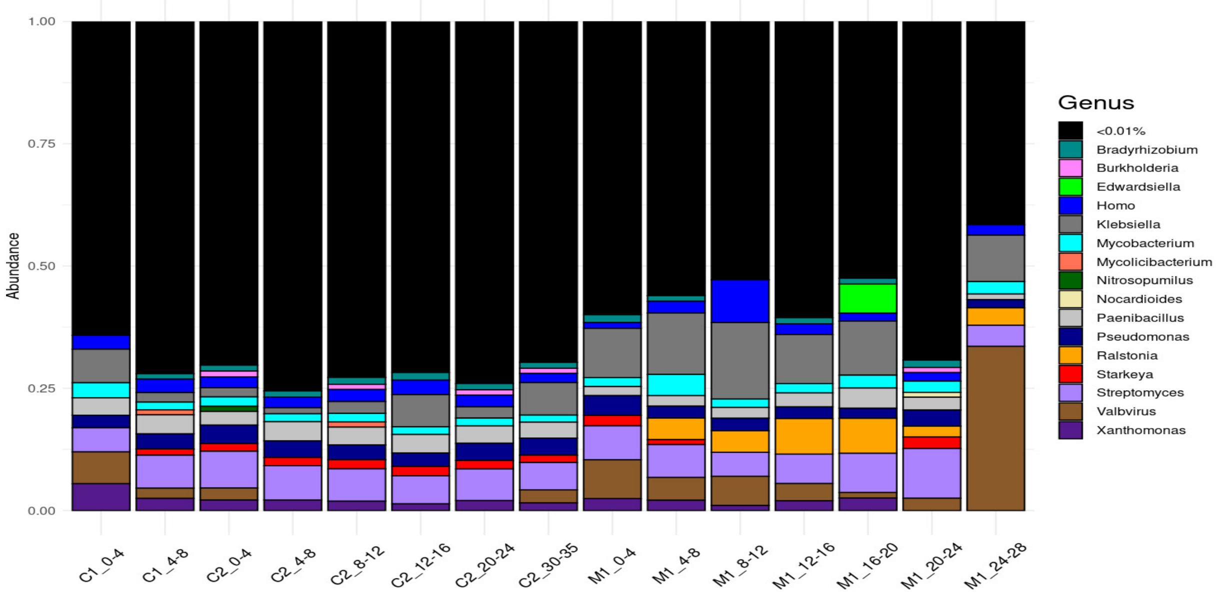

Figure S2: The taxonomic distribution of Genus in the Mediterranea Sea (M1) and Caspian Sea (C1-Core 1, C2-Core 2) sediment samples.

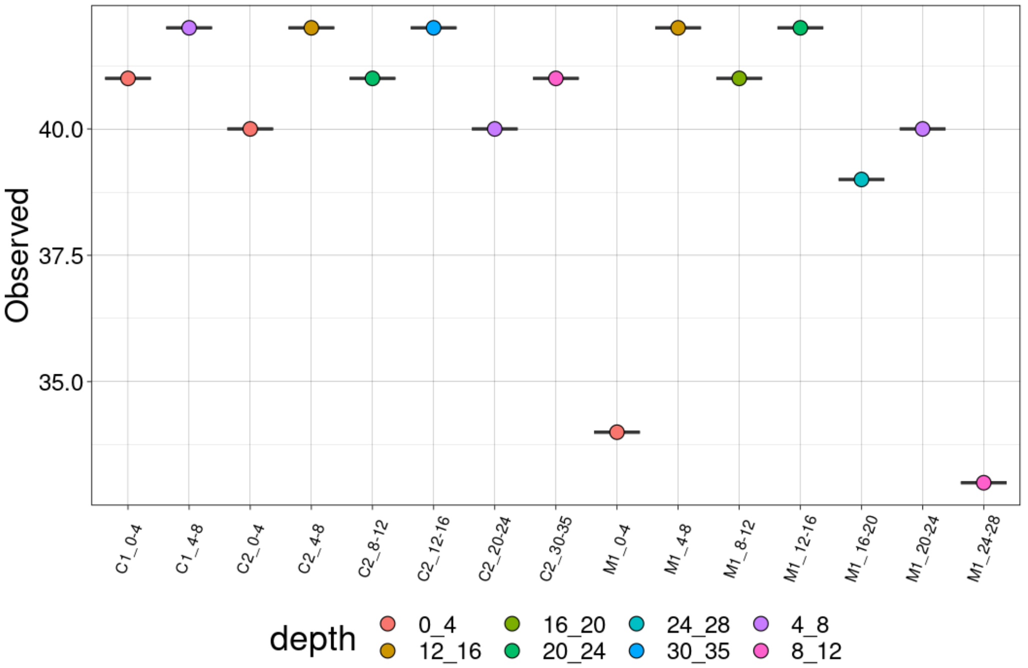

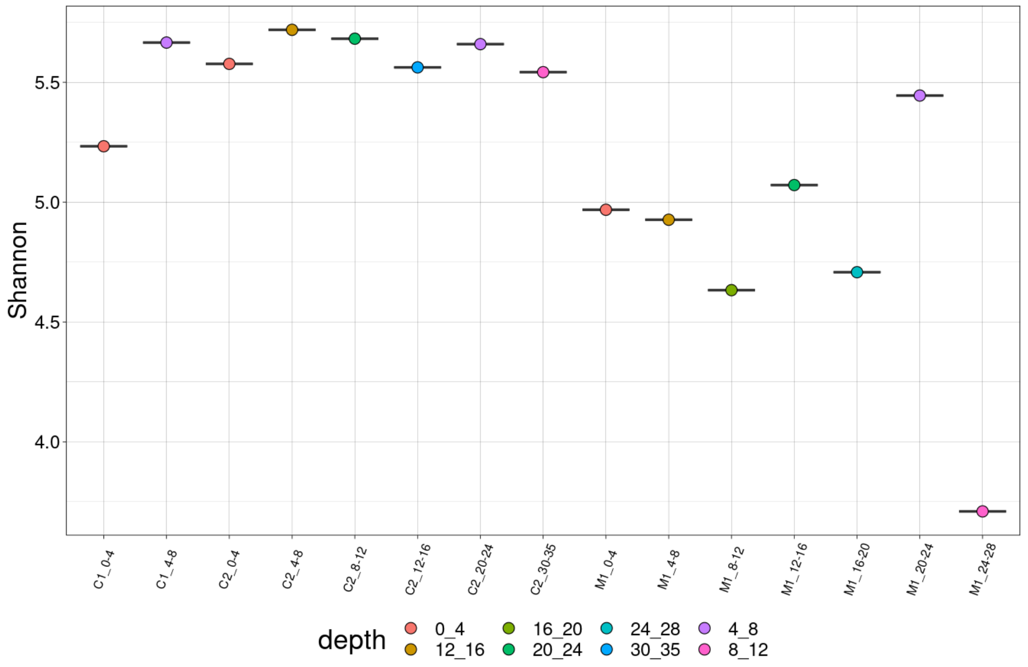

Figure S3. Observed Species Count and Shannon Diversity Index at the Phylum Level in Caspian and Mediterranean Sea Sediments

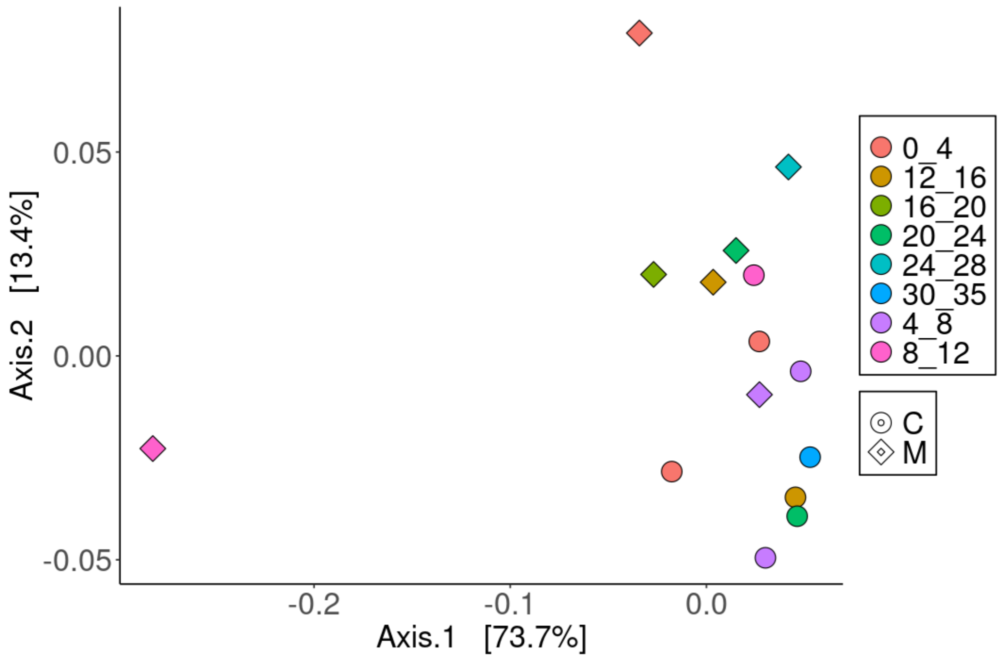

Figure S4: Principal Coordinates Analysis (PCoA) of Microbial Communities at Phylum Level in the Caspian and Mediterranean Sea Sediments

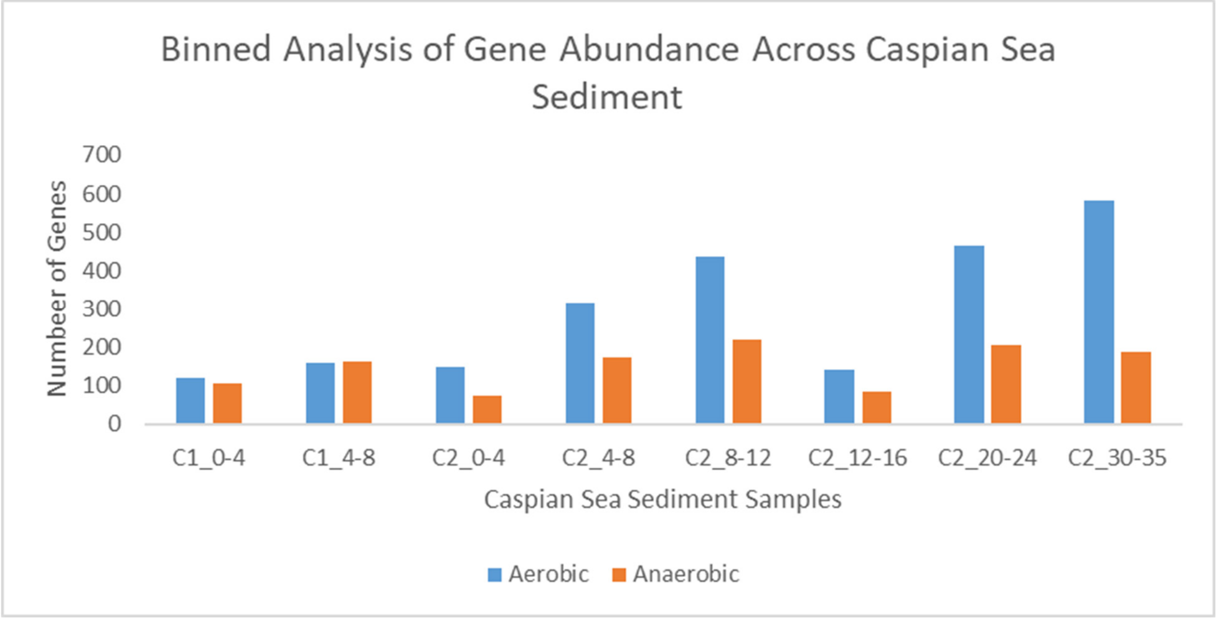

Figure S5: Binned Analysis of Gene Abundance in Caspian Sea Sediment Samples

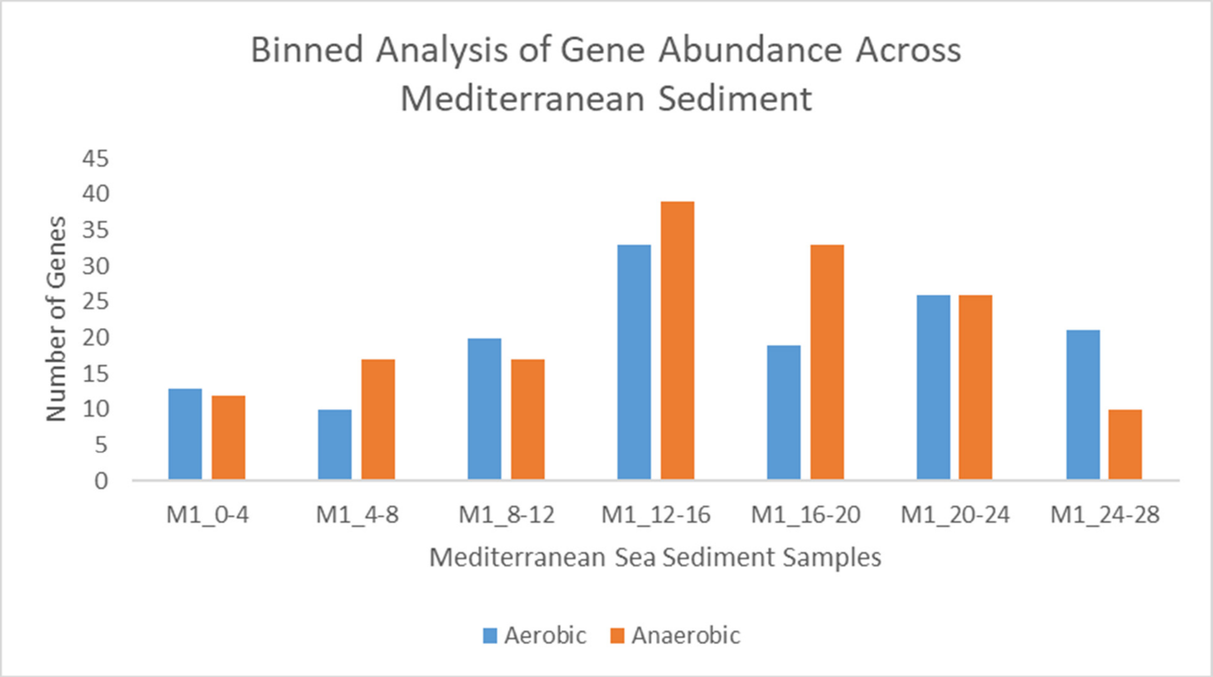

Figure S6: Binned Analysis of Gene Abundance in Mediterranean Sea Sediment Samples

Table S1: List of Aerobic and Anaerobic genes

**Name of Substrate**	**Enzyme Name**	**Gene Name**	**Category**	**Type**
Aerobic Hydrocarbons				
Aliphatic				
Propane	Propane 2-monooxygenase	prmA/prmC	Aerobic Hydrocarbons	Aliphatic
Alkane C5-C13	Alkane hydrolase	AlkB	Aerobic Hydrocarbons	Aliphatic

Alkane C5-C14	Alkane oxidizing cytochrome P450	CYP153	Aerobic Hydrocarbons	Aliphatic
Alkane C20-C35	Flavin-binding alkane monooxygenase	AlmA_GroupI/AlmA_Group III	Aerobic Hydrocarbons	Aliphatic
Alkane C15-C35	Long-chain alkane hydrolase	LadA_alpha/LadA_beta/LadB	Aerobic Hydrocarbons	Aliphatic
Aromatic				
Toluene	Toluene-4-monooxygenase	TmoA_BmoA/TmoB_BmoB/TmoE	Aerobic Hydrocarbons	Aromatic
Phenol/Toluene	Phenol/toluene monooxygenase/hydroxylase	TomA1/TomA3/TomA4	Aerobic Hydrocarbons	Aromatic
Phenol/Toluene	Phenol/toluene 2-monooxygenase	DmpO	Aerobic Hydrocarbons	Aromatic
Phenol	Phenol hydroxylase		Aerobic Hydrocarbons	Aromatic
Naphthalene	Benzene/toluene/naphthalene dioxygenase subunit	MAH_alpha/MAH_beta/NdoB/NdoC	Aerobic Hydrocarbons	Aromatic
Naphthalene	Similar to benzene/toluene/naphthalene dioxygenase subunit	non_NdoB_type	Aerobic Hydrocarbons	Aromatic
Dibenzothiophene	Dibenzothiophene monooxygenase	DszC	Aerobic Hydrocarbons	Aromatic
Anaerobic Hydrocarbons				
Aliphatic				

n-Alkane	Alkylsuccinate synthase	AssA	Anaerobic Hydrocarbons	Aliphatic
n-Alkane	Molybdopterin-family alkane C2 methylene hydroxylase	AhyA	Anaerobic Hydrocarbons	Aliphatic
Aromatic				
Ethylbenzene	Molybdopterin-family ethylbenzene dehydrogenase subunit alpha	CmdA	Anaerobic Hydrocarbons	Aromatic
2-Methyl-naphthalene	Naphthylmethyl succinate synthase		Anaerobic Hydrocarbons	Aromatic
Benzene	Benzene carboxylase		Anaerobic Hydrocarbons	Aromatic
Naphthalene	Naphthalene carboxylase		Anaerobic Hydrocarbons	Aromatic
Benzene/Toluene	Benzylsuccinate synthase	BssA	Anaerobic Hydrocarbons	Aromatic
